# Evaluation of Adipokines, Inflammatory Markers, and Sex Hormones in Simple and Complex Breast Cysts' Fluid

**DOI:** 10.1155/2016/5174929

**Published:** 2016-05-12

**Authors:** Paweł Madej, Grzegorz Franik, Piotr Kurpas, Aleksander Owczarek, Jerzy Chudek, Magdalena Olszanecka-Glinianowicz

**Affiliations:** ^1^Department of Endocrinological Gynecology, Medical Faculty in Katowice, Medical University of Silesia, 40-752 Katowice, Poland; ^2^Department of Statistics in Sosnowiec, Medical University of Silesia, 40-752 Katowice, Poland; ^3^Pathophysiology Unit, Department of Pathophysiology, Medical Faculty in Katowice, Medical University of Silesia, 20-093 Katowice, Poland; ^4^Health Promotion and Obesity Management Unit, Department of Pathophysiology, Medical Faculty in Katowice, Medical University of Silesia, 40-752 Katowice, Poland

## Abstract

*Objective*. The aim of the study was to analyze the association between levels of adipokines in the breast cyst fluid and in the circulation in relation to the type of cysts.* Material and Measurements*. A cross-sectional study involved 86 women with breast cysts (42 with simple cysts and 44 with complex cysts). Plasma and breast cyst fluid leptin, adiponectin, visfatin/NAMPT, resistin, TNF-*α*, and IL-6 levels, in addition to serum levels of estradiol, progesterone and prolactin, and anthropometric parameters and body composition (by bioimpedance method), were measured.* Results*. The levels of leptin, adiponectin, and resistin were significantly lower in breast cyst fluid than in plasma regardless of the cyst type. Contrarily, the levels of visfatin/NAMPT and TNF-*α* were significantly increased, and IL-6 levels were similar in the breast cyst fluid and plasma in both study groups. There was no correlation between corresponding levels of leptin, adiponectin, resistin, visfatin/NAMPT, TNF-*α*, and IL-6 in breast cyst fluid and plasma.* Conclusions*. Higher levels of visfatin/NAMPT and TNF-*α* in the fluid from simple and complex breast cysts than in plasma suggest that their local production is related to inflammation.

## 1. Introduction

Fibrocystic disease in women is a benign breast disease, also known as mastopathy, cystic degeneration or breast fibrocystic dysplasia that constitutes approximately 55% of benign breast lesions [1].

The disease occurs in 7% of women, usually about 40 years old, and detected cysts are often multiple and bilateral. The pathological changes in the breast involve connective and fat tissues, epithelium lining ducts, and glandular follicles with a tendency to the formation of fluid-filled cysts. It is long-standing disease that may occur with or without cell proliferation and atypia and predispose to breast cancer development. The risk of breast cancer in these subjects is 2–4 times higher than in the general population [2].

The established method of differentiating cysts from solid masses is breast sonography. This method distinguishes simple cysts with 100% accuracy and is sufficient to establish the diagnosis [3]. The characteristics of the simple cyst include an anechoic, well-circumscribed mass with an imperceptible wall and posterior acusonic enhancement [4]. The breast lesions that do not meet all these criteria are defined as complex, atypical, or complicated cysts [5]. A complex breast cyst is characterized by a presence of internal echoes, thin septations, an intracystic mass, or a perceptible wall or by the absence of definitive posterior wall enhancement [4].

The pathogenesis of fibrocystic disease has not been fully explained. An excessive cell proliferation of follicular epithelium of the milk ducts, the growth of connective tissue stroma, and the formation of cysts is mostly a consequence of abnormal lobular involution or enlargement of the occluded ducts [6]. Absolute or related-to-the-progesterone deficiency hyperestrogenism is considered as one of main etiological factors of the disease. Hormone replacement therapy seems to protect against fibrocystic disease in postmenopausal women [7].

The fibrocystic disease is diagnosed on the basis sonography (sonomammography), mammography, cytological and biochemical assessment of the breast cyst fluid, and histopathology [8, 9]. In particular, the fine-needle aspiration cytology or biopsy are recommended in the complex breast cysts due to their potential malignancy [5].

Hormones, electrolytes, and mitogenic proteins, present in the breast cyst fluid, suggest the increased risk for cysts progression to neoplastic lesions [9, 10]. Among factors predisposing for breast cancer development there are obesity and circulating adipokines [9–15].

However, so far there is the lack of studies that assessed the prognostic importance of adipokines levels in the breast cyst fluid.

The aim of this study was to analyze the association between levels of adipokines in the breast cyst fluid and in the circulation in relation to the type of cysts.

## 2. Material and Methods

The cross-sectional, prospective study involved 86 women with fibrocystic disease, [42 with simple cysts (BI-RADS 2) and 44 complex cysts (BI-RADS 3 or 4)] aged 25–45 years, with stable body mass for the last 3 months. The exclusion criteria included pregnancy, lactation, menopause, acute and chronic diseases, using any pharmacological therapy, and addiction to smoking and/or alcohol. In addition, on the basis of fine-needle aspiration cytology, the women with the suspicion of malignant cysts were excluded from the study. Study protocol was approved by the Bioethical Committee of Medical University of Silesia, and each participant gave the informed consent.

All study subjects were examined within 21 and 25 days of menstrual cycle. Anthropometric measurements (body mass and height) were performed and BMI was calculated according to the standard formula. Body composition was assessed by the bioimpedance method using Bodystat 1500 (Douglas, Isle of Man). Venous blood samples (15 mL) were withdrawn in the mornings between 8.00 and 9.00 a.m., after an overnight fast (14 h) and collected according to recommendations of the kits manufacturers. Plasma and serum aliquots were frozen and stored at −70°C.

Breast cyst fluids were obtained by ultrasound-guided needle (G 25–27) aspiration biopsy (LOGIQ 3 PRO and ALOKA PROSOUND SSD-3500 SV equipped with linear 7.5–10 MHz transducer). Centrifuged cyst fluid aliquots were frozen and stored at −70°C.

### 2.1. Laboratory Procedures

Serum prolactin (PRL), estradiol (E2), and progesterone were determined by ECLIA (Roche Diagnostic GmbH, Mannheim, Germany) with a lower limit of sensitivity <0.05 ng/mL, <5 pg/mL, and 0.03 ng/mL, respectively, and interassay coefficients of variations were <4.0%, <4.6%, and <2.4%, respectively.

ELISA method was used for measurements of plasma and breast cyst fluids leptin (TECOmedical AG, Sissach, Switzerland), adiponectin (TECOmedical AG, Sissach, Switzerland), resistin (R&D, Minneapolis, MN, USA), visfatin/NAPMT (BioVendor, Brno, The Czech Republic), TNF-*α* (R&D, Minneapolis, MN, USA), IL-6 (R&D, Minneapolis, MN, USA), with the lower limit of sensitivity of 0.2 ng/mL, 0.06 *μ*g/mL, 0.05 ng/mL, 0.03 ng/mL, 0.19 pg/mL, and 0.1 pg/mL, respectively; intra- and interassay coefficients of variations were <6.8% and <7.7% for leptin, <4.7% and <6.7% for adiponectin, <5.5% and <9.2% for resistin, <9.1% and <5.6% for visfatin/NAPMT, <8.7% and 10.4% for TNF-*α*, and <7.8% and 9.6% for IL-6.

### 2.2. Statistical Analysis

Statistical analysis was performed using STATISTICA 9.0 PL (StatSoft, Cracow, Poland) software and R software environment. There was no missing data in the database. The results are presented as mean values ± standard deviation or with median and interquartile range in case of heavy-skewed distribution. Distribution of variables was evaluated by the D'Agostino-Pearson test. Homogeneity of variances was assessed by the Levene test. Quantitative variables were compared with two-way ANOVA with Duncan test post hoc. The assessment of associations between variables was done with the multivariate linear regression and the backward stepwise procedure. Outliers were identified based on Cook's distance values. The Cook-Weisberg test was used to test the residuals for heteroskedasticity. Model calculation was performed, including evaluation of multicollinearity, which was assessed with the variance inflation factor (VIF). The VIF should not exceed more than 5. Goodness of fit of obtained model was assessed with the *F* test and determination coefficient *R* ^2^. All the results were considered as statistically significant with a* p* value of < 0.05.

## 3. Results

The characteristics of women with simple and complex cysts, including anthropometric parameters and hormones levels, are presented in Table 1.

The frequency of females that gave births, and with past abortions, as well as the visceral obesity, was similar in both groups (75.5% versus 63.6%; *p* = 0.33 and 13.2% versus 27.3%; *p* = 0.14; and 56.6% versus 45.4%; *p* = 0.38, resp.).

The serum estradiol, progesterone, and prolactin levels did not differ between women with simple and complex cysts (Table 1).

The plasma leptin level was significantly higher in the group with simple cysts than in those with complex cysts. However, leptin concentrations in the breast cyst fluid did not differ between the study groups (Table 2).

The breast cyst fluid levels of leptin, adiponectin, and resistin were significantly decreased compared to plasma in both study groups. Contrarily, levels of visfatin/NAMPT and TNF-*α* in breast cyst fluid were significantly increased in relation to plasma in both study groups. In turn, IL-6 levels in breast cyst fluid and plasma were similar in both study groups (Table 3).

### 3.1. Correlations between Breast Cyst Fluid and Plasma Adipokines Levels

There were no correlations between corresponding breast cyst fluid and plasma levels of leptin, adiponectin, visfatin/NAMPT, resistin, TNF-*α*, and IL-6 and between the size of the cyst and plasma adipokines levels.

### 3.2. Multiple Linear Regression Backward Stepwise

There were no statistically significant factors explaining variability of leptin, adiponectin, visfatin/NAMPT, resistin, and TNF-*α* levels in breast cyst fluid in multiple, backward stepwise linear regression analysis, while IL-6 level in breast cyst fluids decreased by serum estradiol levels (*β* = −0.005; *p* < 0.05).

The results of multiple, backward stepwise linear regression revealed that plasma leptin levels increased with higher BMI and waist circumference values (*β* = 1.44; *p* < 0.05 and *β* = 0.009; *p* < 0.05, resp.), as well as fat mass (*β* = 0.014; *p* < 0.05). Plasma resistin levels increased with age (*β* = 0.07; *p* < 0.05). Plasma TNF-*α* levels increased with fat mass (*β* = 0.045; *p* < 0.05) and decreased with higher estradiol levels (*β* = −0.0021; *p* < 0.05). There were no statistically significant factors influencing plasma IL-6 levels.

## 4. Discussion

Our study revealed the lack of differences in estradiol, progesterone, and prolactin levels between women with simple and complex cysts. So far there is the lack of studies assessing the differences between serum sex hormones levels in women with simple and complex cysts. Our study did not analyze sex hormones levels in the breast cyst fluids. Previously markedly higher oestrogens and progesterone levels were detected in cyst fluid compared to the blood [10]. In addition, it has been suggested that estrone and progesterone levels in breast cyst fluid are markers of breast cancer development [16]. The role of sex hormones in the pathogenesis of fibrocystic disease was confirmed by study showing that hormone replacement therapy prevents its development [7].

The plasma leptin level was significantly higher in the subgroup with simple cysts than that with complex cysts, corresponding to a slightly, however not significantly greater, BMI, waist circumference, and fat mass. It should be noted that in contrast to an association observed in postmenopausal women, we did not observe correlation between serum estrone and estradiol concentration and plasma leptin levels [17]. However, in our study leptin concentrations in the breast cyst fluid did not differ between study subgroups and were several times lower than in the circulation. This suggests that leptin penetrates slightly from the circulation to both simple and complex cysts. It should be stressed that there is the lack of experimental studies assessing the role of leptin in oncogenic transformation of complex breast cysts. However, an experimental study showed that leptin may induce proliferative response in breast cancer cells but not in the normal breast cells [11]. In addition, it has been observed that knockdown of leptin decreases tumor volume and number of metastases to the lung and liver [18]. Moreover, the synergy between the leptin/Ob-Rb/STAT3 signaling pathway and the HER2 receptor is the mechanism that inhibits effect of tamoxifen by differential regulation of apoptosis-related genes [19]. Furthermore, expression of leptin was greater in the breast tissues adjacent to the tumor compared to tumor specimens [20]. The last observation partially supports a hypothesis that leptin from the circulation may stimulate breast cysts development. However, it is not known whether leptin participates in the oncogenic transformation of fibrocystic disease. As in the general population, it has been shown that circulating leptin levels in cystic fibrosis are associated with percentage of body fat [21]. Thus, the role of leptin in the oncogenic transformation of cystic fibrosis requires further studies, especially including obese women with cystic fibrosis as well as normal weight and obese control groups.

Similar observations concern plasma and breast cyst fluid levels of adiponectin and resistin. Our data suggest that these adipokines are not synthetized by the cysts and only slightly penetrate from the circulation into the cyst fluid. The lack of correlation between these adipokines levels in plasma and breast cyst fluid suggests a variable, case specific diffusion through the cyst wall. It should be mentioned that high resistin expression in breast cancer tissue is associated with a more malignant clinic pathological status and poor patient survival [22]. So far there is the lack of experimental and clinical studies assessing the impact of leptin, adiponectin, and resistin on the risk of cancer development in women with breast cysts. Therefore, the prediction of the risk for breast cancer development on the basis of cyst fluid level is doubtful. Further studies with long-term follow-up are necessary to explain this issue.

Contrary to leptin, resistin, and adiponectin, IL-6 levels were similar and the levels of visfatin/NAMPT and TNF-*α* in breast cyst fluid were significantly greater than in the circulation in both study subgroups. These data suggest that their local production within the cyst is related to mild inflammation. It is unclear whether inflammation is a pathogenic factor involved in the development of cysts or formed as secondary reaction. It has been suggested that IL-6 and TNF-*α* have a local “protector” role in gross cystic disease and may be used as a marker to identify cyst type [23]. However, contrary to this study [23] we did not observe association between TNF-*α* and IL-6 in breast cyst fluids, but in accordance we found a negative association between TNF-*α* and estradiol levels. In addition, we found an inverse relation between IL-6 and estradiol levels in breast cyst fluid. Our results may suggest anti-inflammatory effect of estrogen and the lack of induction of aromatase expression by TNF-*α*, described in breast cancer [24], in the breast cyst.

Furthermore, the increased levels of visfatin/NAMPT in breast cyst fluid show that induction of expression of* NAMPT* is not only a treatment of breast cancer tissue [25], but already characterized benign lesions, breast cysts. Overexpression of visfatin/NAMPT in epithelial cells has been shown to induce epithelial to mesenchymal transition, a morphological and functional switch that confers cancer cells an increased metastatic potential. This mechanism is independent of NAMPT enzymatic activity and NAMPT product nicotinamide mononucleotide. Visfatin/NAMPT activates the TGF*β* signaling pathway by increased TGF*β* production [25]. Thus, on the basis of our results and previously published studies we suggest that visfatin/NAMPT concentration in breast cyst fluid but not in the circulation may be a potential marker for stratification of the risk of cancer development. This is in accordance with the results obtained by Assiri et al. [26] in plasma samples of premenopausal women with breast cancer, while in postmenopausal women with breast cancer circulating visfatin/NAMPT levels were increased [26, 27]. In addition, it was recently suggested that circulating leptin, resistin, and visfatin levels have some diagnostic values for breast cancer in postmenopausal women [28]. This observation has to be verified by further studies. Of interest we observed that circulating visfatin/NAMPT levels were increased in elderly women with components of metabolic syndrome and decreased with age; however, the difference between subjects with and without metabolic syndrome was small [29].

The presented study has some limitations related to its cross-sectional design (the lack of follow-up) and a small percentage of overweight and obese women. In addition, we did not assess the levels of heparin sulfate and plasmin activity in breast cysts fluid that may reflect the function of epithelial cells forming cysts [30, 31].

In conclusion higher levels of visfatin/NAMPT and TNF-*α* in the fluid from simple and complex breast cysts than in plasma suggest that their local production is related to inflammation.

## Figures and Tables

**Table 1:** The characteristics of study groups.

	Women with simple cyst *N* = 42	Women with complex cyst *N* = 44	*p*
Age [years]	38 ± 7	37 ± 6	NS
Body mass [kg]	62.9 ± 10.9	61.7 ± 12.4	NS
BMI [kg/m^2^]	23.5 ± 4.2	22.5 ± 3.8	NS
Fat [%]	37.3 ± 7.4	35.3 ± 5.7	NS
Fat mass [kg]	23.9 ± 8.0	22.2 ± 8.0	NS
Waist circumference [cm]	82.8 ± 12.3	80.7 ± 12.8	NS
Estradiol [pg/mL]	91.1 ± 60.4	94.7 ± 65.7	NS
Progesterone [ng/mL]	1.0 ± 0.3	1.1 ± 0.3	NS
Prolactin [ng/mL]	223.0 ± 101.2	225.9 ± 88.2	NS

**Table 2:** Plasma and specimens from the cysts adipokines levels in study subgroups.

	Women with simple cyst *N* = 42	Women with complex cyst *N* = 44	*p*
Plasma leptin [ng/mL]	15.1 (8.5–21.9)	8.0 (4.6–14.6)	<0.05
Cyst fluid leptin [ng/mL]	1.1 (0.8–1.6)	0.8 (0.5–1.5)	NS
Plasma adiponectin [*μ*g/mL]	3.4 (2.0–6.4)	3.8 (1.9–4.6)	NS
Cyst fluid adiponectin [*µ*g/mL]	0.03 (0.02–0.06)	0.03 (0.02–0.04)	NS
Plasma visfatin/NAPMT [ng/mL]	1.4 (0.7–2.4)	1.4 (0.9–3.0)	NS
Cyst fluid visfatin/NAPMT [ng/mL]	9.1 ± 2.3	8.3 ± 1.6	NS
Plasma resistin [ng/mL]	8.6 ± 1.9	8.4 ± 1.9	NS
Cyst fluid resistin [ng/mL]	1.0 ± 0.6	1.0 ± 0.6	NS
Plasma TNF-*α* [pg/mL]	1.2 ± 0.6	1.0 ± 0.5	NS
Cyst fluid TNF-*α* [pg/mL]	3.5 ± 1.5	3.4 ± 1.5	NS
Plasma IL-6 [ng/mL]	2.0 ± 1.2	1.5 ± 0.9	NS
Cyst fluid IL-6 [ng/mL]	2.0 ± 1.3	1.7 ± 1.5	NS

**Table 3:** The comparison of plasma and specimen adipokines levels in study subgroup.

	Women with simple cyst			Women with complex cyst		
	*N* = 42			*N* = 44		
	Plasma	Cyst fluid	*p*	Plasma	Cyst fluid	*p*
Leptin [ng/mL]	15.1 (8.5–21.9)	1.1 (0.8–1.6)	<0.001	8.0 (4.6–14.6)	0.8 (0.5–1.5)	<0.001
Adiponectin [*μ*g/mL]	3.4 (2.0–6.4)	0.03 (0.02–0.06)	<0.001	3.8 (1.9–4.6)	0.03 (0.02–0.04)	<0.001
Visfatin/NAPMT [ng/mL]	1.4 (0.7–2.4)	9.1 ± 2.3	<0.001	1.4 (0.9–3.0)	8.3 ± 1.6	<0.001
Resistin [ng/mL]	8.6 ± 1.9	1.0 ± 0.6	<0.001	8.4 ± 1.9	1.0 ± 0.6	<0.001
TNF-*α* [pg/mL]	1.2 ± 0.6	3.5 ± 1.5	<0.01	1.0 ± 0.5	3.4 ± 1.5	<0.01
IL-6 [ng/mL]	2.0 ± 1.2	2.0 ± 1.3	NS	1.5 ± 0.9	1.7 ± 1.5	NS
